# Sorption and degradation processes of imidacloprid in Florida soils

**DOI:** 10.1371/journal.pone.0305006

**Published:** 2024-09-06

**Authors:** Qudus O. Uthman, Davie M. Kadyampakeni, Jorge A. Leiva, Jonathan D. Judy, Peter Nkedi-Kizza

**Affiliations:** 1 Soil, Water and Ecosystem Sciences Department, University of Florida, Gainesville, Florida, United States of America; 2 Department of Crop and Soil Sciences, North Carolina State University, Raleigh, North Carolina, United States of America; 3 Soil, Water, and Ecosystem Sciences Department, University of Florida, Citrus Research and Education Center, Lake Alfred, Florida, United States of America; 4 School of Agronomy, University of Costa Rica, Guanacaste Campus, Liberia, Costa Rica; Indian Council of Agricultural Research - Indian Agricultural Research Institute, INDIA

## Abstract

Imidacloprid (IDP) is an active ingredient of the Admire brand pesticide used to control the vector (Asian citrus psyllid) that transmits the causative organism *Candidatus Liberibacter asiaticus (CLas*) for citrus greening or huanglongbing disease. Imidacloprid products are applied via soil drench where citrus roots are mostly concentrated which is between 0 and 60 cm depth. These soil depths exhibit different characteristics that may affect IDP leaching beyond the rooting zone. Representative soil samples were collected from Entisols and Ultisols, which are the dominant soil orders under citrus production in central Florida, at 15 cm increments up to 60 cm to estimate and understand the batch sorption, kinetics, equilibria, and degradation of IDP. Results showed that the equilibrium time for IDP at 0–15 cm depth (10 hours) was 2 times faster than at 15–60 cm (20 hours) for the Entisol. Nevertheless, all depths reached equilibrium within 24 hours for the Entisol. The 0–30 cm depth adsorbed 2 times more IDP than the 30–60 cm depth for both soils. Nevertheless, the adsorption coefficient was approximately ≤ 1 mL g^-1^ for both soils. The half-life of IDP in both soils ranged from 10 to 17 days. The Entisol showed higher adsorption than the Ultisol at both depths, probably due to relatively lower organic carbon (OC) content in the Ultisol compared to the Entisol. Thus, the Ultisol showed high IDP leaching vulnerability compared to the Entisol. Movement of IDP is affected by the amount of OC in the citrus critical zone.

## Introduction

Best management practices for citrus production include pesticide application such as imidacloprid (IDP) to control disease vectors and nutrient management to maintain fruit production [1]. Citrus production in central Florida most often occurs on Entisols and Ultisols [2]. Entisols are soils that have little to no horizon development [3]. They usually lack distinct layers (or horizons) other than the original parent material [3]. These soils are often found in areas with recent deposition of parent material, like floodplains, steep slopes, sand dunes, or volcanic ash falls [3]. Due to their lack of horizon development, many Entisols have limited natural fertility and may require significant inputs for agricultural use [3]. However, some, like the Fluvents found in floodplains, can be quite fertile [4]. Ultisols are well-developed soils that have a subsurface horizon in which clays have accumulated, often with significant iron oxide content [5]. They have a clear topsoil and subsoil division. Ultisols typically form in older, stable landscapes, especially in warm, humid climates [5]. They tend to be acidic and can be quite leached, with many essential nutrients washed out of the topsoil [5]. They may require liming and other amendments for successful agriculture [6]. The citrus critical zone for both soil orders is the 0–60 cm soil depth where the roots are most concentrated for water and nutrient uptake [7]. Soil horizons occurring in this zone are A (rich in organic matter) and E (mostly siliceous sands) (Soil Survey Staff, 2023).

**Fig 1:**
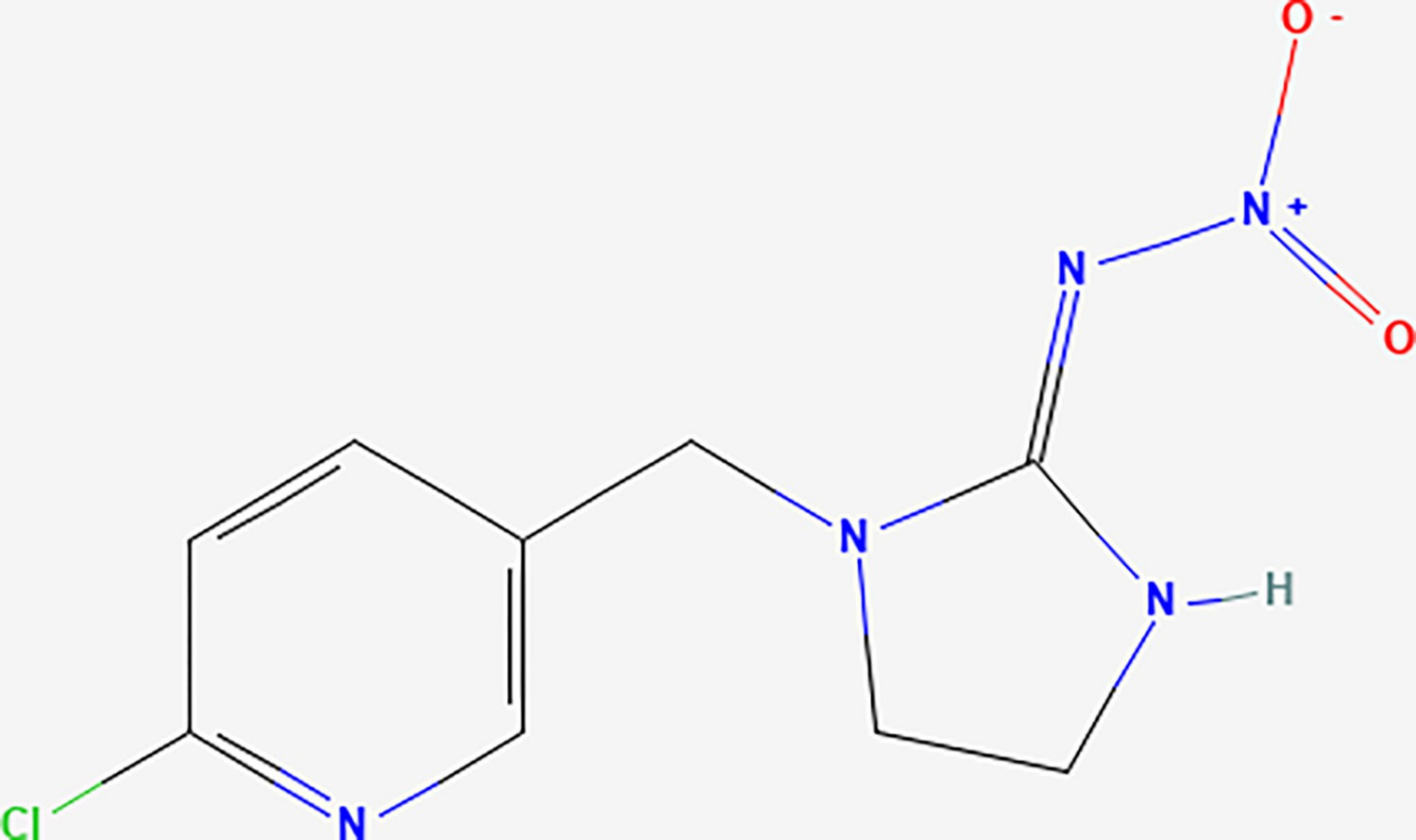
2-dimensional chemical structure of Imidacloprid (IUPAC name: 1-(6-chloro-3-pyridylmethyl)-N-nitroimidazolidin-2-ylideneamine) with a molecular formula of C9H10ClN5O2 (confirm from the product itself) (National Center for Biotechnology Information, 2022).

Imidacloprid (IDP) is a neonicotinoid insecticide, a synthetic derivative of nicotine [8]. It is a zwitterionic polar compound that has both negative and positive charge (Fig 1). It does not volatize, is soluble in water, and can be taken up by plant roots from the soil solution [9]. It is an insecticide that kills Asian citrus psyllids (ACP)–a citrus disease vector that transmits *Candidatus Liberibacter asiaticus* (CLas), causing the citrus greening disease–while also being toxic to beneficial insects such as bees [10]. Factors affecting IDP soil adsorption include pH, cation exchange capacity (CEC), and the nature of the soil clay fraction [11], but organic matter and clay mineral type are the dominant factors affecting IDP sorption in soils [11,12]. Imidacloprid may also diffuse into the soil organic matter fraction after it has undergone adsorption-desorption processes [9,13].

The K_OC_ is the partition or adsorption coefficient normalized to the soil organic carbon (OC) content that determines the influence of OC sorption in soils [14]. The influence of OC can be found in several studies where K_OC_ < 1000 mL g^-1^ [11,12,15,16]. Imidacloprid in Immokalee fine sand has a K_OC_ between 13–230 mL g^-1^, whereas it barely degrades with a half-life between 359–948 days [9,17]. Since ACP could develop resistance to IDP, which would reduce its control impact, it is important to revisit the application of IDP in soil of this nature (well-drained and sandy in texture, with very low organic matter <0.5%). This may lead to concerns about groundwater contamination and pollution. The objectives of this study are to: 1) estimate and compare the batch sorption kinetics and equilibria processes of IDP in the Entisol and Ultisol and 2) determine the half-life of IDP in the Entisol and Ultisol.

## Materials and methodology

### Soil samples collection and site description

A series of subsamples from multiple positions were collected at four depths in 15 cm increments using a soil auger and composited for two sites located at the Citrus Research and Education Center (CREC) in Lake Alfred, FL with latitude and longitude coordinates of 28°06’24.53” N, 81°42’51.10” W (Entisol) and 28°06’58.91” N, 81°42’42.30” W (Ultisol). Both sites have elevations of 47 m and 49 m above sea level with slopes of 0% and 3.9% for the Entisol and Ultisol, respectively. The Entisol is classified as hyperthermic, uncoated Lamellic Quartzipsamments while the Ultisol is a loamy, siliceous, subactive, hyperthermic Grossarenic Paleudults (Soil Survey Staff, 2023). A soil profile description for those sites was performed to match the horizon identification claimed by the official soil series description.

Soil samples were collected from each horizon for mineralogical analysis (Tables 1 and 2). The difference between the two soils was the presence of illuvial clay material in the deeper layers of the Ultisol. However, the citrus critical zone at 0–60 cm essentially has the same soil horizons (A and E) with less than 95% sand for the two soils. Horizon A is the surface horizon with a high amount of organic matter while horizon E is the subsurface that has undergone eluviation of clay materials. The OC content was determined using the Walkley-Black method [18]. The cation exchange capacity was calculated as the sum of all cation elements (acid plus basic cation elements) extracted using Mehlich III extracting solution and determined by inductively coupled plasma (ICP) [19,20].

**Table 1:** Average cation exchange capacity (CEC), organic carbon (OC) content, and soil pH in water of Entisol and Ultisol with standard error.

Soil	Depth	CEC	OC	pH in water^†^
	cm	cmol_c_ kg^-1^	g 100 g^-1^ of soil	
Entisol	0–15	4.52 ± 0.19	0.25 ± 0.03	7.43 ± 0.06
Entisol	15–30	3.37 ± 0.15	0.13 ± 0.02	7.54 ± 0.07
Entisol	30–45	2.74 ± 0.10	0.10 ± 0.02	7.38 ± 0.13
Entisol	45–60	2.41 ± 0.07	0.10 ± 0.01	7.30 ± 0.11
Ultisol	0–15	4.03 ± 0.12	0.19 ± 0.02	7.63 ± 0.05
Ultisol	15–30	2.93 ± 0.14	0.12 ± 0.03	7.52 ± 0.09
Ultisol	30–45	2.51 ± 0.10	0.09 ± 0.03	7.35 ± 0.12
Ultisol	45–60	2.21 ± 0.07	0.06 ± 0.01	7.27 ± 0.13

† 1:2 (w/v) soil/water ratio.

**Table 2:** Identification of mineral types in Entisol and Ultisol across four depths.

Soil	Depth, cm	Minerals^†^
Entisol	0–15	Quartz and Kaolinite
Entisol	15–30	Quartz and Kaolinite
Entisol	30–45	Quartz and Kaolinite
Entisol	45–60	Quartz and Kaolinite
Ultisol	0–15	Quartz, Kaolinite, and Hydroxy-interlayered Vermiculite (HIV)
Ultisol	15–30	Quartz, Kaolinite, and Hydroxy-interlayered Vermiculite (HIV)
Ultisol	30–45	Quartz and Kaolinite
Ultisol	45–60	Quartz and Kaolinite

^†^The order of mineral prevalence is from left to right.

### Mineralogical analysis

Air-dried soil samples from each horizon were prepared for identification of clay-sized minerals via X-ray powder diffractometry (XRD). The clay-sized fraction of each sample was isolated by first dispersing the soil sample 1 M NaCl and then centrifuging the sample at 2000 rpm for 5 min, after which the suspended clay particles were decanted. This process was repeated until the supernatant was clear, after which the collected suspension was filtered through a 0.45 μm membrane filter. Then, either 25 mL of MgCl_2_ or KCl (one slide of each was prepared for each sample) was washed through the already mounted filter, followed by 25 mL of deionized water to remove excess salt. The clay particles were then transferred onto a glass slide and analyzed via XRD using an Ultima IV XRD (Rigaku, Tokyo, Japan). Then, each sample was subjected to a series of treatments, as needed, to provide additional information. The MgCl_2_ slide was misted with a glycerol solution and re-scanned, whereas the KCl slide was heated at 110, 200 and 550°C, with the sample being cooled and re-analyzed in between each heat treatment [21].

### Soil sorption batch, kinetics, and equilibria

Since the main compound of interest was IDP, the sorption isotherm model and equilibration time were determined using a chemical non-equilibrium kinetic model as described below. A 2000 mg L^-1^ IDP stock solution was prepared in methanol. The stock solution was used to prepare 1, 3, 5, 7, and 9 μg IDP mL^-1^ in 0.01 M CaCl_2_. 10 mL of solution was added to 10 g of air-dried soil samples in three replicates. Shaking was done with varied time of 2, 8, 16, 32, and 64 hours using an orbital shaker. Thus, there were 15 total polypropylene centrifuge tubes with soil sample for each depth that contained 5 varied IDP concentrations in 3 replicates for each shaking time.

Samples were shaken using Vevor orbital shaker (Houston, Texas, United States) at 180 revolution per minutes (rpm). Samples were centrifuged for 30 minutes at 4000 rpm and supernatant solution was filtered using 0.45 μm pore diameter syringe filter. IDP was analyzed using Agilent 1260 Infinity high performance liquid chromatography (HPLC) with ultraviolet (UV) detector and a LiChrospher reverse phase (RP) select B column (125 × 4.0 mm; Sigma-Aldrich Co.). The mobile phase was 40% HPLC grade water and 60% HPLC grade methanol with an injection volume of 20 μL, a flow rate of 1 mL min^−1^, and a detection wavelength of 270 nm. IDP analytical standard with 99.9% purity from Sigma Aldrich was used in this study. Imidacloprid concentration in the soil was determined using a calibration curve of known concentration between blank samples (0 ppm) and 20 ppm of IDP with 0.99975–0.99998 R^2^.

### Sorption models and theory

**Fig 2:**
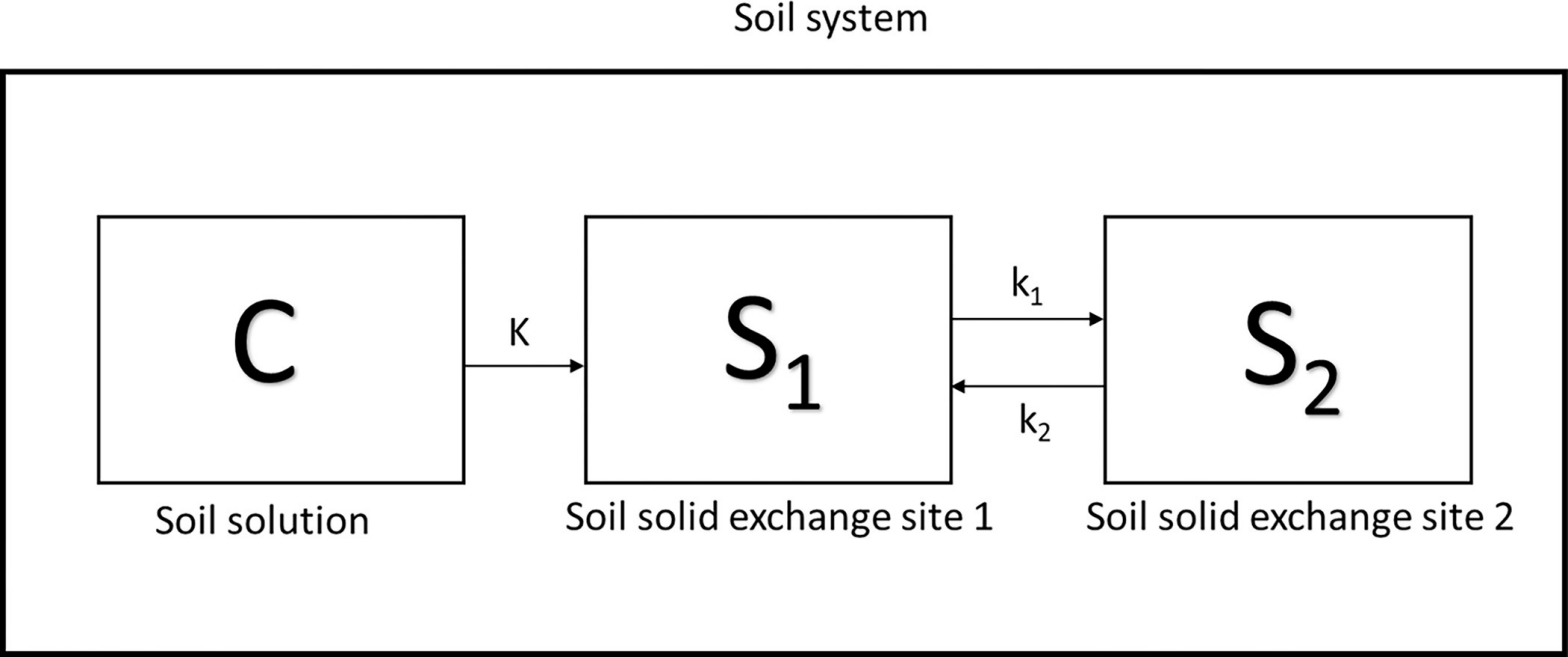
Schematic representation of two-sites nonequilibrium chemical processes in a soil system.

Consider a soil system that is comprised of solid particles that have surface charges for cation exchange and a solution phase (C). It was assumed that the air component of the soil did not participate in the cation exchange reaction. The Entisol and Ultisol were less sandy in texture compared to Spodosols with relatively high percentage of clay at the citrus critical rooting zone [13]. However, clay aggregates might have been mistaken for sand particles, thus, it was hypothesized that there are two chemically non-equilibrium sites. The chemical non-equilibrium and amount of IDP partitioned into the soil could be affected by both clay mineral and OC content. The solid particle phase was divided into two sites: instantaneous (S_1_) and kinetic (S_2_) [22]. The equilibrium linear sorption or partition coefficient is K, the kinetic transfer rate from S_1_ to S_2_ is k_2_, and that from S_2_ to S_1_ is k_1_ (Fig 2) [22].


$$S=S_{1}+S_{2}$$ (1)

$$\frac{S_{1}}{S}=F;\frac{S_{2}}{S}=1-F$$ (2)

$${S=KC;\;S}_{1}=FKC;\;S_{2}=(1-F)KC$$ (3)

Where S is total amount solute in the solid particle surfaces per unit weight of soil (μg g^-1^), F is the fraction of sorption instantaneous sorption site., and C is the equilibrium concentration of solute in the solution phase (μg mL^-1^).

At a time before transfer of solute from S_2_ to S_1_ or vice-versa i.e., 0≤t<t_1_, S_2_ = 0 and C = C_t0_. At that time, it was assumed that no transfer had taken place between the two sites.

$$M_{t}=VC_{t0}+MS_{1}$$ (4)

$$C_{t0}=\frac{M_{t}}{V+FMK}$$ (5)

Where M = mass of air-dried soil (g); M_t_ = total mass of solute in the soil system (μg); V is the volume of the solution added (mL).

At a time when the reaction is at equilibrium i.e., t_2_≤t<t_∞_, C = C_t∞_. At that time, it was assumed the amount of solute per unit weight of soil at the kinetic site does not change with time anymore, $\frac{\delta S_{2}}{\delta t}=0$.

$$M_{t}=VC_{t\infty }+MS_{1}+MS_{2}$$ (6)

$$C_{t\infty }=\frac{M_{t}}{V+MK}$$ (7)

$$\frac{\delta S_{2}}{\delta t}=k_{1}S_{1}-k_{2}S_{2}=0;\;k_{1}=\frac{k_{2}(1-F)}{F}$$ (8)

At a time when there is transfer of solute between the two sites i.e., t1<t<t2, C = C_t_.

$$S_{2}=\frac{M_{t}-MFKC_{t}-VC_{t}}{M}$$ (9)

$$\frac{\delta S_{2}}{\delta t}=k_{1}S_{1}-k_{2}S_{2}$$ (10)

Substitute S_2_ using Eqs (9) in (10)

$$\left(V+MFK)\frac{\delta C_{t}}{\delta t}=k_{2}(M_{t}-C_{t}(V+MK)\right)$$ (11)

$$\int_{0}^{t}\frac{k_{2}dt}{V+MFK}=\int_{C_{t}}^{C_{t0}}\frac{k_{2}dC_{t}}{V+MFK}$$ (12)

Solution to Eq (12) is given as

$$\left(\frac{M_{t}}{V+MK}-C_{t0}\right)e^{-\frac{k_{2}}{\beta }t}=\frac{M_{t}}{V+MK}-C_{t}$$ (13)

Substitute Eqs (7) in (13)

$$C_{t}=C_{t\infty }+(C_{t0}-C_{t\infty })e^{-\frac{k_{2}}{\beta }t}$$ (14)

Let $R=1+\frac{M}{V}K\;and\;\beta =\frac{1+F(\frac{M}{V})K}{R}$; since *M*_*t*_ = *VC*_0_, then $C_{t0}=\frac{C_{0}}{\beta R}\;and\;C_{t\infty }=\frac{C_{0}}{R}$

Our final working two-site nonequilibrium working model will be:

$$\frac{C_{0}}{C_{t}}=\frac{1}{R}+\left(\frac{1}{\beta R}-\frac{1}{R}\right)e^{-\frac{k_{2}}{\beta }t}$$ (15)

If F = 0, then one site nonequilibrium working model will be

$$\frac{C_{0}}{C_{t}}=\frac{1}{R}+\left(1-\frac{1}{R}\right)e^{-\frac{k_{2}}{\beta }t}$$ (16)

The bootstrapping method was used to determine 95% confidence intervals of the parameters [23].

### Soil degradation

About 10 μg g^-1^ of IDP was added to 10 g of air-dried soil sample in triplicates and homogenized. Samples were loosely covered and kept in the dark in cupboard boxes. Moisture content was monitored by weight difference every two weeks, with samples kept at field capacity by replenishing the weight lost with HPLC water. Imidacloprid was extracted using 20% water and 80% methanol mixture after 14, 30, 90, 180, and 310 days. Samples were shaken for 24 hours using orbital shaker. The described methods were carried out on two soils (Entisol and Ultisol) at the citrus critical zone of 0–60 cm soil depths at 15 cm increments. Then, the data were fitted into first order degradation process below in Eq (17)

$$S_{t}=S_{0}e^{-kt}$$ (17)

Where $S_{t}=\frac{v}{m}C_{t}$ is the amount of solute per unit weight of soil (μg g^-1^); S_0_ is the initial amount of solute per unit mass of soil (μg g^-1^); k is the degradation rate constant.

Half-life t_1/2_ is calculated as:

$$t_{1/2}=\frac{0.693}{k}$$ (18)

Shaking was done using the Vevor orbital shaker (Rancho Cucamonga, California, United States). Homogenization was done using the vortex mixer. The matrix container used for this study was polypropylene centrifuge tubes. Each sample was centrifuged for 30 min at 4000 rpm and the supernatant solution was filtered using 0.45μm pore diameter syringe filter. IDP analysis was done using HPLC with UV detector at 270 nm wavelength. The mobile phase was 40% water and 60% methanol. The HPLC grade water and methanol were used for this study. The IDP analytical standard with 99.9% purity from Sigma Aldrich was used for the whole experiment. Imidacloprid concentration in the soil was determined using a calibration curve of known concentration between blank samples (0 ppm) and 20 ppm of IDP with 0.99975–0.99998 R^2^. The bootstrapping method was used to determine 95% confidence intervals of the parameters [23]. Data analysis was done using R and Python programming languages interface [24,25].

## Results

Cation exchange capacity (CEC) and OC decreased as a function of depth for both soils, but the Entisol has greater CEC and OC compared to the Ultisol (Table 1). This may be due to the leaching of clay mineral materials with high specific surface area from the citrus critical zone to the deeper horizon in the Ultisol. The Entisol has 32% more OC than the Ultisol at 0–15 cm depth (Table 1). The 0–15 cm depth has 150% and 90% more OC than the 15–60 cm depth for the Entisol and Ultisol respectively (Table 1). The Entisol has 12% more CEC than the Ultisol at 0–15 cm depth (Table 1). The 0–15 cm depth has 80% and 61% more CEC than the 15–60 cm depth for the Entisol and Ultisol respectively (Table 1).

Mineralogical analysis results showed that both soils are dominated by quartz and kaolinite across all four depths (Table 2). However, hydroxy-interlayered vermiculite (HIV) was identified in the Ultisol’s 0–30 cm sample, in addition to quartz and kaolinite (Table 2). The pH of both soils was consistent, on average about 7.4 across all depths.

**Fig 3:**
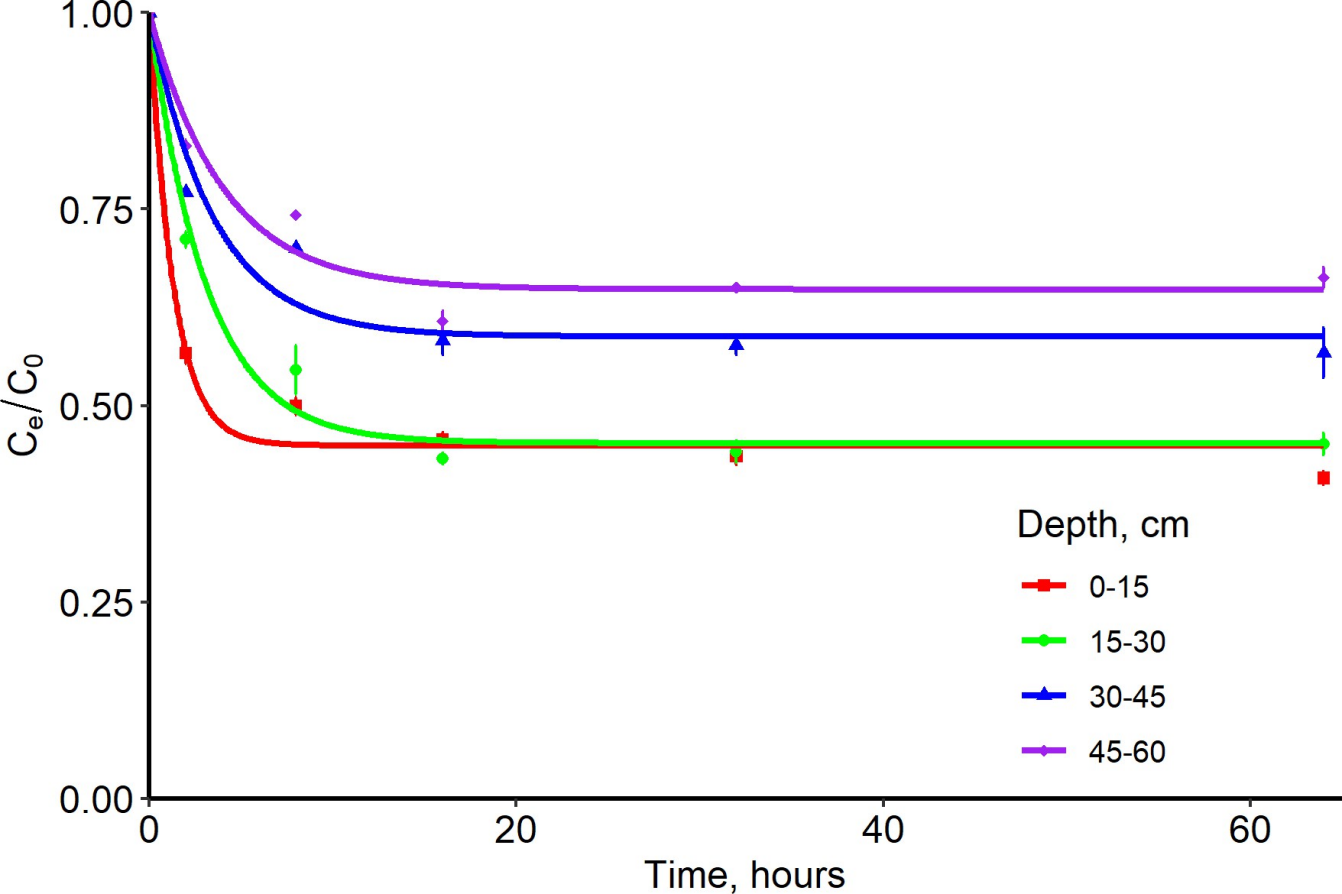
First order reaction kinetics (one site nonequilibrium) of Imidacloprid with an Entisol at four depths. Observations have standard error bars for three replicates. C_e_/C_o_ is the ratio of equilibrium concentration to initial concentration.

Equilibrium time for IDP at the 0–15 cm depth (10 hours) was 2 times faster compared to 15–60 cm depth (20 hours) for the Entisol. Nevertheless, all depths reached equilibrium within 24 hours (Table 3 and Fig 3).

**Table 3:** Confidence intervals one site nonequilibrium parameters of Imidacloprid reaction kinetics with the Entisol and the Ultisol.

Parameters		Alpha		Sorption coefficient, K_D_		K_OC_,		R^2^	
Units	cm	h^-1^		mL g^-1^		mL g^-1^		--	
Soil	Depth	LL	UL	LL	UL	LL	UL	LL	UL
Entisol	0–15	0.334	0.353	1.222	1.261	488.8	504.4	0.818	0.993
Entisol	15–30	0.138	0.155	1.200	1.240	793.8	820.3	0.908	0.968
Entisol	30–45	0.158	0.196	0.684	0.710	653.6	678.4	0.781	0.902
Entisol	45–60	0.148	0.173	0.539	0.560	515.0	535.1	0.857	0.924
Ultisol	0–15	0.410	0.435	0.851	0.875	457.4	470.3	0.833	0.993
Ultisol	15–30	0.357	0.380	0.702	0.713	575.0	584.0	0.834	0.959
Ultisol	30–45	0.391	0.415	0.410	0.423	440.8	454.7	0.808	0.963
Ultisol	45–60	0.368	0.402	0.340	0.354	531.6	553.5	0.767	0.947

K_OC_: Partition coefficient due to organic carbon content.

LL = Lower limit; UL = Upper limit.

**Fig 4:**
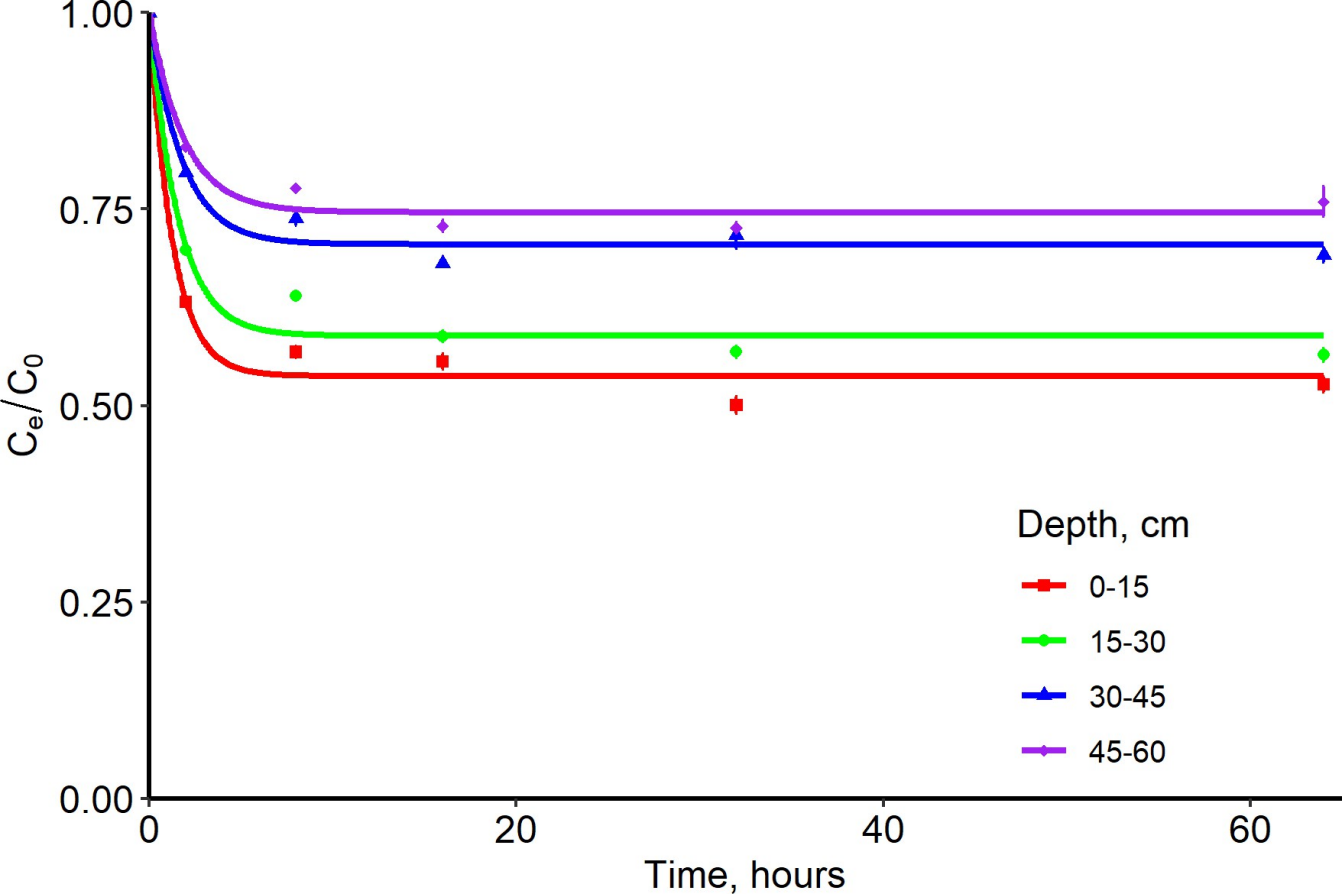
First order reaction kinetics (one site nonequilibrium) of imidacloprid with an Ultisol at four depths. Observations have standard error bars for three replicates. C_e_/C_o_ is the ratio of equilibrium concentration to initial concentration.

**Fig 5:**
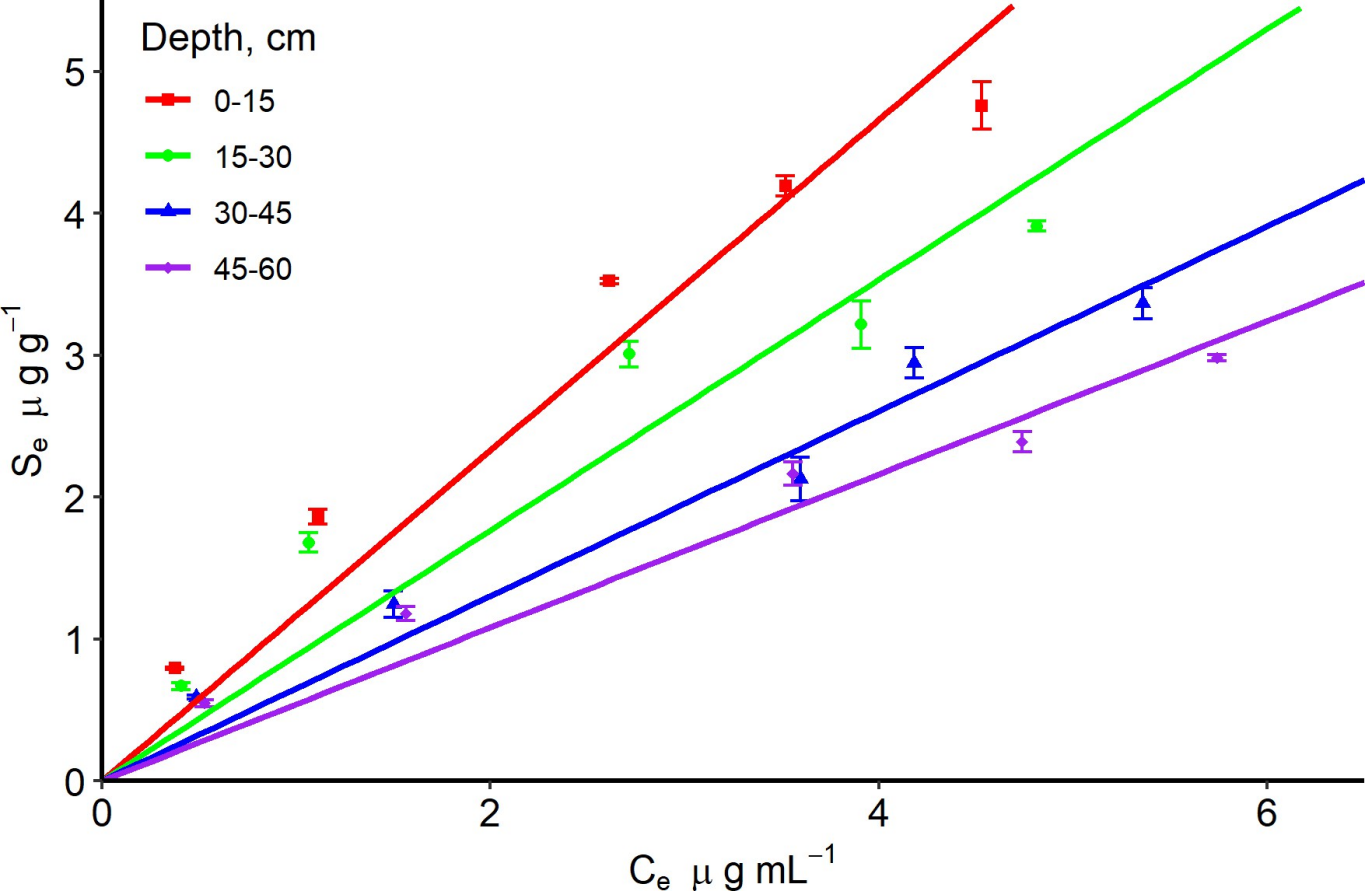
Linear sorption isotherm of imidacloprid with an Entisol at four depths. Observations have standard error bars of three replicates. S_e_ and C_e_ are sorbed and equilibrium concentrations, respectively.

**Fig 6:**
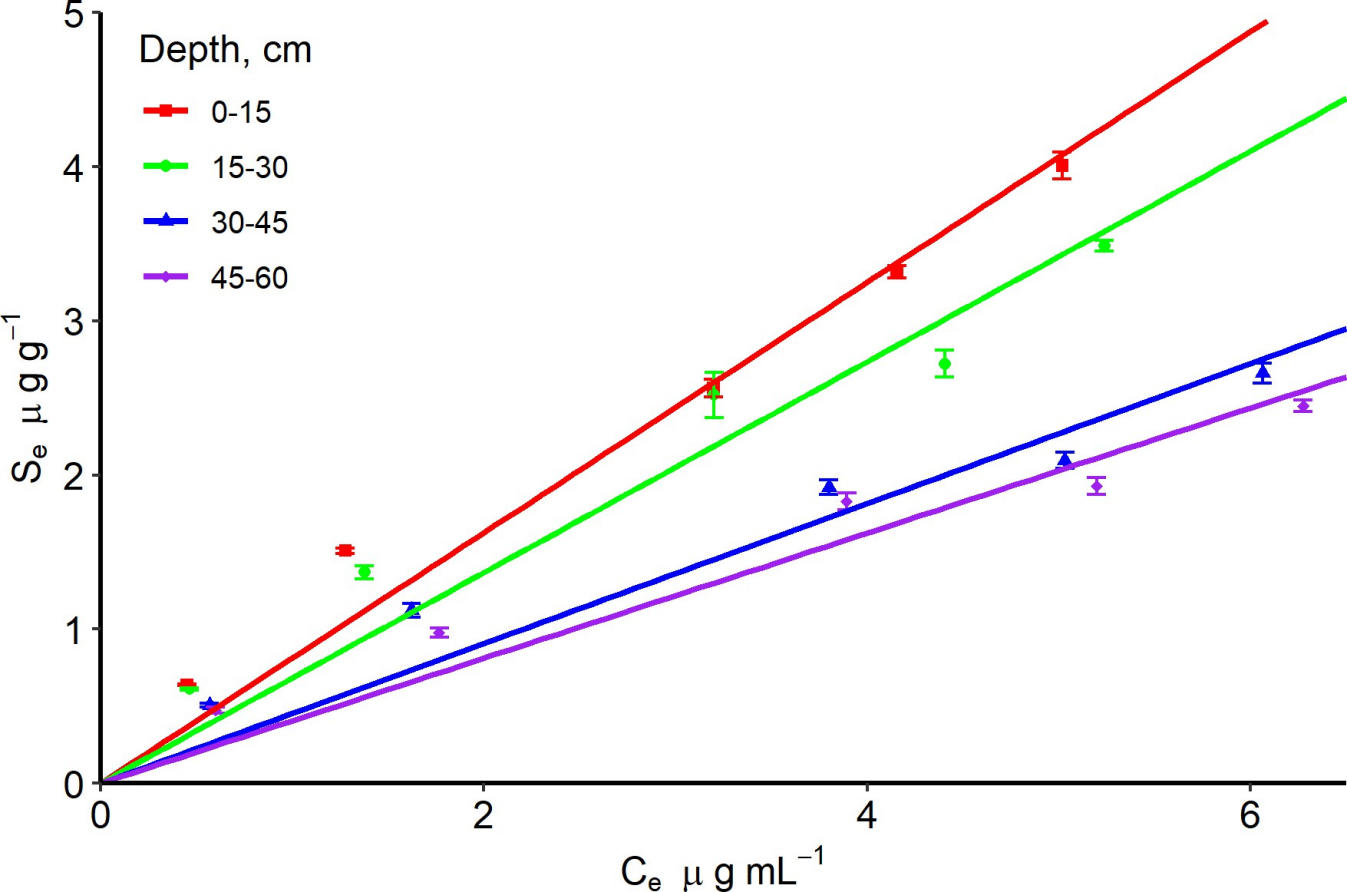
Linear sorption isotherm of imidacloprid with an Ultisol at four depths. Observations have standard error bars of three replicates. S_e_ and C_e_ are sorbed and equilibrium concentrations, respectively.

The mass transfer rate at the 0–15 cm depth was 2 times more than the 15–60 cm depth for the Entisol (Table 3 and Fig 3). This could be attributed to the relatively high OC content (50% greater OC) at 0–15 cm depth. The mass transfer rate and equilibrium time were consistent for all depths in the Ultisol (Table 3 and Fig 4). The 0–30 cm soil depth adsorbed 2 times more IDP than the 30–60 cm soil depth for both soils (Tables 3 and 4; Figs 5 and 6), but the adsorption coefficient was approximately ≤ 1 mL g^-1^ for both soils probably due to the OC content and presence of clay minerals in the 0–60 cm soil depth for both soils.

**Table 4:** Confidence intervals of linear partition coefficient of Imidacloprid in the Entisol and the Ultisol.

Parameters		Sorption coefficient, K_D_		K_OC_		R^2^
unit	cm	mL g^-1^		mL g^-1^		
Soil	Depth	LL	UL	LL	UL	
Entisol	0–15	1.059	1.262	423.6	504.8	0.977
Entisol	15–30	0.778	0.981	514.7	649.0	0.961
Entisol	30–45	0.594	0.703	567.6	671.8	0.979
Entisol	45–60	0.499	0.580	476.8	554.2	0.983
Ultisol	0–15	0.764	0.860	410.7	462.3	0.989
Ultisol	15–30	0.622	0.742	509.4	607.7	0.977
Ultisol	30–45	0.416	0.491	445.1	525.4	0.980
Ultisol	45–60	0.374	0.437	582.1	680.1	0.982

K_OC_: Partition coefficient due to organic carbon content.

LL = Lower limit; UL = Upper limit.

The Entisol showed higher sorption than the Ultisol throughout the depths by 45%, because of the relatively low OC in the Ultisol compared to the Entisol (Table 1). This is consistent with the significant differences in K_OC_ values for both soils at 0–45 cm depth. The K_OC_ for the Entisol was significantly different from the Ultisol at 0–15, 15–30, and 30–45 cm but similar at 45–60 cm when K_OC_ values were compared using confidence intervals (Table 1). Thus, OC might have played a significant role in the movement of IDP beyond the root zone. The half-life of IDP in the Entisol compared to the Ultisol at a specific depth ranged between 10–17 days and were not significantly different from each other since confidence intervals overlap (Table 5). However, the Ultisol has an average half-life 7% more than the Entisol in the critical root zone, which implies that it would take relatively longer time for 50% of IDP to degrade in the Ultisol compared to the Entisol (Table 5).

**Table 5:** Confidence intervals of half-life for Imidacloprid in the Entisol and the Ultisol.

		k, day^-1^		Half-life, days		
Soil	Depth, cm	mean	(LL, UL)	mean	(LL, UL)	R^2^
Entisol	0–15	0.058	(0.055, 0.061)	11.95	(11.36, 12.60)	0.974
Entisol	15–30	0.052	(0.043, 0.065)	13.33	(10.66, 16.12)	0.714
Entisol	30–45	0.053	(0.042, 0.068)	13.08	(10.19, 16.50)	0.562
Entisol	45–60	0.046	(0.041, 0.053)	15.07	(13.08, 16.90)	0.891
Ultisol	0–15	0.053	(0.050, 0.057)	13.08	(12.16, 13.86)	0.965
Ultisol	15–30	0.050	(0.048, 0.051)	13.86	(13.59, 14.44)	0.994
Ultisol	30–45	0.045	(0.041, 0.049)	15.40	(14.14, 16.90)	0.948
Ultisol	45–60	0.049	(0.041, 0.059)	14.14	(11.75, 16.90)	0.784

k: First order degradation constant.

LL = Lower limit; UL = Upper limit.

## Discussion

For a pesticide to be considered under the Persistent Organic Pollutants (POPs) criteria, its half-life should be greater than 6 months in soil or sediment (Stockholm Convention, 2001). Several studies reported long persistence of IDP in soils with half-lives ranging from weeks to years [26]. Leiva et al. (2017) [13] reported persistence of IDP in uncoated sandy (>95% sand) soils with half-life of 0.98–2.60 years compared to this study´s coated sandy (<95% sand) soils with half-life of 10–17 days. Studies with short half-life of IDP were influenced by various factors such as soil properties, temperature, moisture content, pH, organic matter content, and the presence of specific microbial communities [26]. The half-life of 10–17 days for this study was relatively low compared to other studies, but a microbial study was not conducted to determine the factor responsible for the result since microbes are known to attenuate the degradation of IDP in soils [27–29]. The first-order degradation mathematical model demonstrated the best fit compared to all other models evaluated. The relatively short half-life might be attributed to IDP metabolites that were not considered in this study.

The use of mathematical models to explain sorption processes is evident in the relevant literature. Konda et al. (2002) [30] used a multi-step adsorption model to describe sorption processes of IDP in brown forest soils. They found that IDP has a K_D_ ≈ 1 mL g^-1^, which was similar to the result of this study despite our model differences. Zhang et al. (2022) [31] showed that hydrophobic partitioning, cation-π, H-bonding, and p/π-π electron donor-acceptor interactions and electrostatic interactions were the sorption mechanisms of IDP in adsorption with co-precipitated goethite and dissolved OC. Another study found that montmorillonite was active in the sorption of IDP when organic matter was removed from the soil [32]. Similarly, Chen et al. (2022) showed that IDP sorption processes and mechanisms were physisorption and chemosorption. It was reported that hydrophobic partitioning and pore-filling interaction played an important role in the sorption of IDP [33]. However, the clay-size mineral fractions in the Entisol and Ultisol used in this study were mainly quartz and kaolinite across all depths. The association of mineral surfaces might have been confused with that of organic colloids [32]. Liu et al. (2006) [11] found that OC was the controlling factor of sorption behavior in six soils, but CEC, clay amount, and type did not contribute much to sorption. Conversely, Fernandez-Bayo and Romero (2002) [34] reported positive correlation of CEC with K_D_ in eight agricultural soils of Europe. Cation exchange capacity of both soils is less than 5 cmol_c_ kg^-1^ (Table 2) and could have contributed to the amount of IDP sorbed. A possible explanation could be that the relatively high OC content (23% greater OC) and presence of kaolinite in the Entisol compared to the Ultisol was responsible for the relatively higher K_D_. Similarly, Jolin et al. (2017) [35] reported the affinity of organic compounds to soil surfaces. The results of this study exhibit a similar sorption mechanism of hydrophobic partitioning of IDP in both soils as evidently supported by literature cited earlier.

Imidacloprid sorption mechanism in Florida sandy soils is dominantly influenced by OC content, an important property for hydrophobic organic chemicals partitioning in the soils [36]. K_OC_ values of IDP for both soils are similar with the values reported in several studies [11,31,32,36]. An important environmental concern of IDP soil application is the leaching vulnerability on both soils. Soil OC has a significant influence on the movement of IDP beyond the citrus critical zone of 0–60 cm depths. IDP would move faster beyond the citrus critical zone due to less OC content. Mineral colloids of both soils could help to retain nutrients in the citrus critical zone depending on the amount and the colloidal properties, but OC is critically important to retain IDP in the citrus critical zone long enough to degrade.

## Conclusion

Imidacloprid had a low adsorption coefficient for both soils, but the Ultisol indicates higher leaching potential or other fates such as plant uptake or degradation, compared to the Entisol. This study revealed that there was presence of kaolinite and 23% more OC in the 0–60 cm soil depth of the Entisol compared to the Ultisol. The half-life of IDP for both soils ranged from 10 to 17 days. Nevertheless, environmental caution needs to be exercised when applying IDP to both soils. Clay materials, especially organic matter, influence the extent of IDP partitioning in the soils under study. Thus, future studies should evaluate OC quality on sorption process of IDP. Also, farming practices that promote soil OC sequestration and could increase the partitioning of IDP in both soils long enough to promote citrus root uptake and lower the risk for IDP leaching beyond the citrus root zone.
